# A review of the impact of processing on nutrient bioaccessibility and digestion of almonds

**DOI:** 10.1111/ijfs.13192

**Published:** 2016-07-31

**Authors:** Myriam Marie‐Louise Grundy, Karen Lapsley, Peter Rory Ellis

**Affiliations:** ^1^ Diabetes and Nutritional SciencesKing's College LondonFranklin‐Wilkins Building, 150 Stamford StreetLondonSE1 9NHUK; ^2^ Almond Board of California1150 Ninth Street Ste.1500ModestoCA95354USA

**Keywords:** Almonds, dietary fibre, bioaccessibility/digestibility, lipids, processing effects

## Abstract

Almond kernels contain phytochemicals and nutrients that potentially have positive health benefits in relation to heart disease, diabetes and obesity. One important mechanism associated with these benefits is an imposed limit on bioaccessibility (release) of nutrients, such as lipids, from almond tissue during mastication and digestion. Recent studies have demonstrated the importance of food structure during the digestion of plant foods. In particular, in the almond kernel, depending on its structure and degree of processing, the amount of lipid released from the almond tissue matrix and the fatty acids produced from lipolysis has been found to vary substantially. This review aims at discussing the commercial methods of almond processing and the different almond forms produced for human consumption, mainly with respect to their impact on nutrient composition, digestion and metabolism.

## Introduction

Almond seeds or kernels are highly versatile and can be eaten on their own or as part of a number of food products. Almonds are consumed world‐wide with the United States being the largest producer (Gradziel, 2011; Harris & Ferguson, 2013). There is a wide range of methods currently used to process almond seeds (e.g. heat processing and particle size reduction). These processes have led to the development of almond‐based products with enhanced organoleptic characteristics, but this is not without consequences for the nutritional properties of the almond tissue.

From a nutritional perspective, almonds are a useful food and ingredient for other foods as they contain a range of macro‐ and micronutrients as well as phytochemicals. Epidemiological evidence and the results of numerous metabolic studies in humans have shown that the consumption of almonds and other nuts reduce a number of risk factors associated with noncommunicable disease, notably type 2 diabetes, obesity and cardiovascular disease (Richardson *et al*., 2009; Tan & Mattes, 2013; Nishi *et al*., 2014; Berryman *et al*., 2015). One crucial factor that seems to explain these putative health benefits is the physical behaviour of almonds in the gastrointestinal (GI) tract, especially how almonds are disassembled and the rate and extent to which they release macronutrients such as lipid. However, mechanisms that explain the physiological effects and the long term benefits of tree nuts like almonds are not well understood, particularly the properties of almond cell walls in each compartment of the GI tract (i.e. mouth, stomach and intestine). Obtaining information about the changes occurring to the structure of the almond tissue as the digestion process progresses and the mechanisms of lipid release is of crucial importance (Ellis *et al*., 2004; Mandalari *et al*., 2014; Grundy *et al*., 2015a,b). For instance, the size and microstructure of the particles following oral processing have a significant effect on nutrient bioaccessibility (release), digestion kinetics and other physiological processes in the GI tract (Grundy *et al*., 2015a). The purpose of this review is to present the most common processing techniques applied to almond kernels, and how they affect almond structure and their subsequent impact on the digestion of lipid and other nutrients.

## Almond anatomy and composition

### Macrostructure

**Figure 1:**
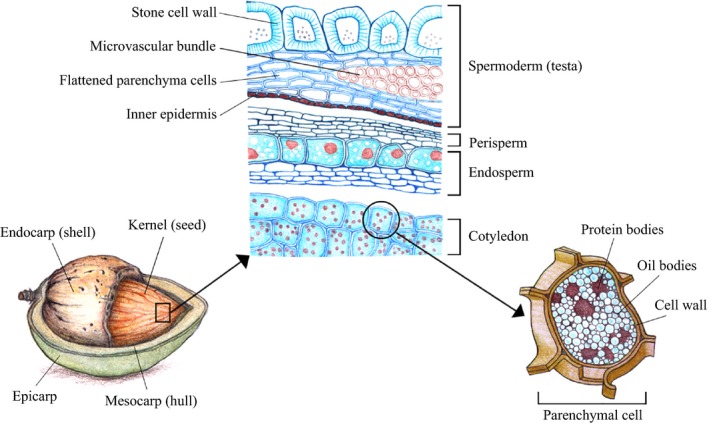
Multiscale structure of almond fruit with kernel. Note that the size of the almond cell is about 35 μm and the oil body between 1 and 5 μm.

The sweet almond *(Prunus dulcis* (Mill.) D.A. Webb or *Amygdalus communis* L.) belongs to the *Rosaceae* family. Almond is a drupe^1^ of which the only edible part is the kernel or seed (Gradziel, 2011). The latter is composed of an embryo (two cotyledons), surrounded by a skin also called testa. The pericarp, which encloses the kernel, contains a green fleshy hull and a hard pitted shell (Fig. 1).

### Microstructure and composition

**Figure 2:**
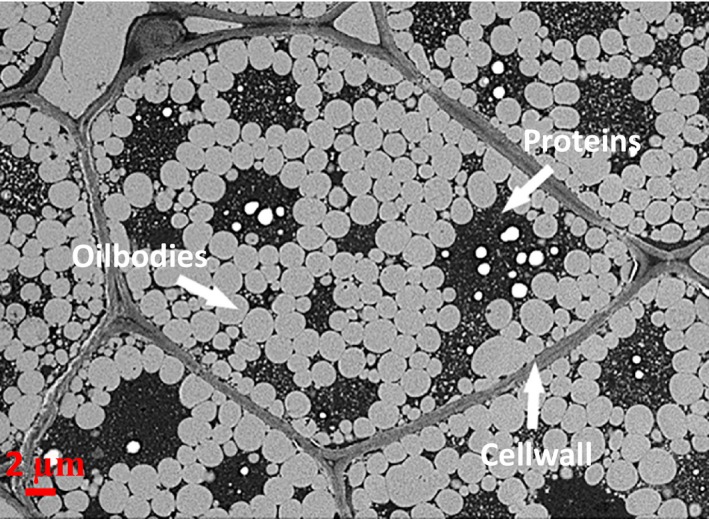
Transmission electron micrograph image of almond kernel showing oil bodies (white inclusions), protein bodies (black inclusion) and the cell walls. Scale bar = 2 μm.

The almond cotyledons (i.e. the white lipid‐bearing tissue) are made of rounded cells, principally parenchyma, with a relatively thin cell wall (~0.1–0.3 μm) (Fig. 2). Pigmented sclerenchyma (outer layer) and parenchyma cells as well as xylem tissue compose the testa (Mandalari *et al*., 2010a). The testa cells possess a secondary cell wall, which is confirmed by the presence of a significant amount of lignin (Femenia *et al*., 2001). A layer of aleurone cells, containing globoid crystals as well as protein and lipid bodies, forms the endosperm that separates the testa (spermoderm and perisperm) from the cotyledon (Winton & Winton, 1932; Young *et al*., 2004).

Almonds are a valuable dietary source of lipid (comprising mainly monounsaturated fatty acids), protein, dietary fibre, vitamins (e.g. vitamin E), minerals, phenolic compounds and phytosterols (Bolling *et al*., 2011; Yada *et al*., 2011; Fernandez‐Cuesta *et al*., 2012) (Table 1).

**Table 1:** Nutrient and total phenolic composition of almonds

	Ranges per 100 g of almond	
	g	mg
Macronutrients		
Protein	16–23	
Lipid	44–61	
Saturated fats	3–4	
Monounsaturated fats	31–35	
Polyunsaturated fats	11–12	
Carbohydrates		
Total sugars	4–6	
Total dietary fibres	11–14	
Water	4–5	
Micronutrients		
Minerals		
Calcium		264–300
Magnesium		230–268
Phosphorus		440–510
Potassium		705–730
Zinc		3.0–4.1
Copper		0.9–1.3
Manganese		1.2–1.8
Vitamins		
Riboflavin		1.0–1.1
Vitamin E (α‐tocopherol)		25–27
Total phenolic compounds		260–350

Adapted from (Richardson *et al*., 2009; Bolling *et al*., 2011; Yada *et al*., 2011, USDA, 2015).

#### Protein

The major storage protein found in almonds, sometimes called amandin or almond major protein, belongs to the legumin class of seed proteins, which itself is a part of the globulin family (Osborne & Campbell, 1896; Kshirsagar *et al*., 2011). Globulin proteins are classified according to their sedimentation coefficient, with the legumin type being 11S. Amandin accounts for about 70% of the total soluble proteins. It has a hexameric structure and each of the six subunits is composed of two polypeptides (α‐chain of about 45 kDa and β‐chain of about 20 kDa) linked by a disulphide bridge, with a molecular weight of approximately 450 kDa (Sathe *et al*., 2002). Along with 2S albumin, amandin, in particular the glutamine‐rich region of the protein, is responsible for the food allergy reactions observed in certain individuals following the consumption of almonds (Alasalvar & Shahidi, 2009; Willison *et al*., 2013).

#### Lipids

Almond lipids, composed predominantly of triacylglycerols (TAG), are assembled into oil bodies. These organelles are delimited by a monolayer of phospholipids in which oleosins, integral proteins, are embedded (Tzen *et al*., 1993; Beisson *et al*., 2001b). Compared with other tree nuts, the almond lipid has a low amount of saturated fatty acids, but nonetheless it contains a significant proportion of poly‐ and monounsaturated fatty acids, with oleic acid being the predominant fatty acid (Robbins *et al*., 2011). Thus, depending on the harvest and variety, the kernel is made of approximately 50% of lipids of which 70–80% is oleic acid, 15% linoleic acid and 5% palmitic acid (Yada *et al*., 2011).

#### Carbohydrates and dietary fibre

The contents of available carbohydrates (i.e. mostly sugars) and dietary fibre (i.e. cell walls) in almond kernels are about 5.5% and 11.8%, respectively (Ellis *et al*., 2004). Little is known, however, about the structural organisation of the almond cell walls in any part of the kernel. Also, as highlighted by our group (Grassby *et al*., 2014) and others (McDougall *et al*., 1996; Waldron *et al*., 2003), each cell type of edible plant tissues, including almond tissue, has a distinct cell wall composition. Furthermore, the precise molecular composition and spatial arrangement of the polysaccharides and noncarbohydrates in almond cell walls have not been completely delineated. A number of compositional studies have found that the cell walls of almond kernel cotyledon, following hydrolysis of the cell wall polysaccharides and analysis by gas‐liquid chromatography, are rich in arabinose, uronic acid, glucose, xylose and galactose, which implies that the cell wall is composed of arabinose‐rich polysaccharides, including pectic material (Femenia *et al*., 2001; Dourado *et al*., 2004; Ellis *et al*., 2004). The cell walls of almond testa contain arabinose, galacturonic acid, glucose, xylose and galactose, but their proportions are different to those in the cotyledon and mannose, rhamnose and fucose are also part of their composition (Ellis *et al*., 2004; Mandalari *et al*., 2010a).

#### Micronutrients and phytochemicals

The almond kernel is rich in vitamins and minerals, and is considered as a good source of vitamin E (tocopherols), riboflavin, calcium, magnesium, phosphorus, potassium, zinc, copper and manganese (Rodushkin *et al*., 2008; Richardson *et al*., 2009) (Table 1). Almonds also contain a wide variety of phenolic compounds, mainly proanthocyanidins, flavonoids and phenolic acids (Perez‐Jimenez *et al*., 2010; Bolling *et al*., 2011; Xie *et al*., 2012), which are located predominantly in the skin and are responsible for their antioxidant properties (Mandalari *et al*., 2010b). Phytosterols are also found in significant amounts (~270 mg 100 g^−1^) in almond kernels, β‐sitosterol being the predominant type (Fernandez‐Cuesta *et al*., 2012; Alasalvar & Bolling, 2015; Forcada *et al*., 2015). Evidence suggests that the phytosterols reduce blood concentrations of LDL cholesterol and so these compounds may also contribute to the reduced risk of cardiovascular disease associated with consuming almonds (Plat & Mensink, 2005; Berryman *et al*., 2015). Sweet almond contains trace amounts (~0.2 to 16 mg 100 g^−1^ of almond) of amygdalin, a poisonous cyanogenic glycoside, whereas bitter almond has a high level of this glycoside (3300 to 5400 mg 100 g^−1^) (Lee *et al*., 2013).

## Processing techniques and their impact on almond structure

Almonds are consumed predominantly in the raw, sliced or roasted forms, although marzipan as well as almond butter, milk and oil are also commonly found (Wareing *et al*., 2000; Gradziel, 2011). They are principally eaten as a snack but they can contribute to the composition of various sweet (e.g. breakfast cereals, cakes and biscuits) and savoury (e.g. salads, curries and tajines) dishes and food products. According to the Food and Agriculture Organization (FAO), the annual world production of almonds has been estimated to be about 1 930 000 metric tons of shelled product in 2012 (Food and Agriculture Organization of the United Nations, 2012). The main producing countries are the USA (California), Spain, Syria and Italy; California produces ~80% of the world's almonds (Harris & Ferguson, 2013).

The main processing techniques applied to almond and their effect on the structure and the composition of the nut are summarised in Table 2.

**Table 2:** Main processing techniques and their effects on the chemical composition, structure and properties of almonds

Processing	Effect on almond structure and composition	References
Roasting	Water loss Cell wall damage Changes in the cytoplasmic network Loss in oil body integrity (i.e. lipid coalescence) Distortion and aggregation of protein bodies Browning of the almond tissue due to Maillard reaction Lipid uptake (when oil used during roasting)	Altan *et al*. (2011) Grundy *et al*. (2015b) Mandalari *et al*. (2014) Pascual‐Albero *et al*. (1998) Perren & Escher (2013) Varela *et al*. (2006)
Blanching	Alteration in cytoplasmic organisation Skin removal which leads to loss in some micronutrients (e.g. phenolic compounds) Water uptake	Altan *et al*. (2011) Mandalari *et al*. (2010a) Pascual‐Albero *et al*. (1998)
Particle size reduction	Rupture of cell walls particularly on the surface of the almond particle Release of some of the nutrients	Grundy *et al*. (2015a)
Oil extraction	Degradation of the almond tissue to extract the oil Loss in oil body integrity	Gallier *et al*. (2014) Kamal‐Eldin & Moreau (2009)

### Roasting

Roasting is a thermal process that involves dehydration (Perren & Escher, 2013). Almonds can be roasted in different ways (e.g. hot air vs. oil roasting, variations in heating times and duration) to obtain the light, medium or dark roast depending on the colour and moisture content of the resulting almonds. The roasting process has to be performed under well‐defined conditions in order to preserve the almond nutritional properties and prevent off‐flavour formation due to oxidation of unsaturated fatty acids.

The roasted almonds used in our recent studies (Grassby *et al*., 2014; Mandalari *et al*., 2014; Grundy *et al*., 2015a,b, 2016) were provided by the Almond Board of California and were roasted using a two‐step standard procedure of hot air roasting with typical temperatures ranging from ~130 to 154 °C (Almond Board of California, 2007b). The first step employed an intermediate temperature to stabilise the nut microstructure, and the second step was performed at a higher temperature in order to generate the distinctive roasted flavour and brown colour of the cotyledon. Thus, during roasting, some of the moisture is lost by evaporation, and the Maillard reaction takes place, which is a complex reaction between reducing sugars and amino acids and is responsible for the brown colour (Perren & Escher, 2013). This nonenzymic browning enhances the antioxidant capacity of the roasted almond.

The hot air roasting process was shown to lead to very little weight variation in whole almond kernels; most of the loss being attributed to water evaporation (Perren & Escher, 2013). The decrease in water content in roasted almonds has been reported to be between 40.7 to 59.1% of the original moisture content of the raw almonds (Altan *et al*., 2011). However, the oil bodies and the endoplasmic network were largely destroyed, and the volume of extracellular pores enlarged. Roasting can therefore greatly affect the structure of almond cells, the cell walls as well as the intra‐cellular oil bodies (Pascual‐Albero *et al*., 1998; Varela *et al*., 2006; Mandalari *et al*., 2014; Grundy *et al*., 2015a,b). In these studies, roasted almond oil bodies appeared to coalesce to form larger oil droplets than the ones observed in raw almond cells. During oil roasting, similar observations were made, but lipid uptake (ranging from 7.2 to 10.3%) from the oil used during roasting was also found to take place (Altan *et al*., 2011). Moreover, roasting is reported to reduce the polyphenol content of the almond skin and subsequently its antioxidant capacity (Bolling *et al*., 2010).

In terms of its physical behaviour during mastication, roasted almonds were found to be more brittle and crunchy and produced more loose particles postchewing than whole raw almonds (Varela *et al*., 2008; Vickers *et al*., 2014). The attributes of roasted almonds described by Vickers and colleagues are likely to be due to the loss of moisture occurring during the roasting process.

### Blanching

Similar to roasting, the blanching procedure decreases potential contamination, such as bacterial and mould growth, and consists of a thermal process that removes almond skin, using either wet or dry methods (Wareing *et al*., 2000). One of the wet methods used consists in peeling off the almond skins after the kernels are bathed in water at 85–100 °C for 2–5 min (Almond Board of California, 2007a). Kernels are dried by hot air, and then cooled down to room temperature. As highlighted above, almond skin is rich in flavonoids and other phenolic compounds, which confer the skin's antioxidant properties. Therefore, removing the skin reduces some of the nutritional attributes of the almond kernel (Garrido *et al*., 2008). Compared with roasted almonds, blanched almonds have a greater water content (Vickers *et al*., 2014). Both the roasting and blanching processes have been demonstrated to have no effect on the allergenicity of almond proteins (Venkatachalam *et al*., 2002).

### Particle size reduction

**Figure 3:**
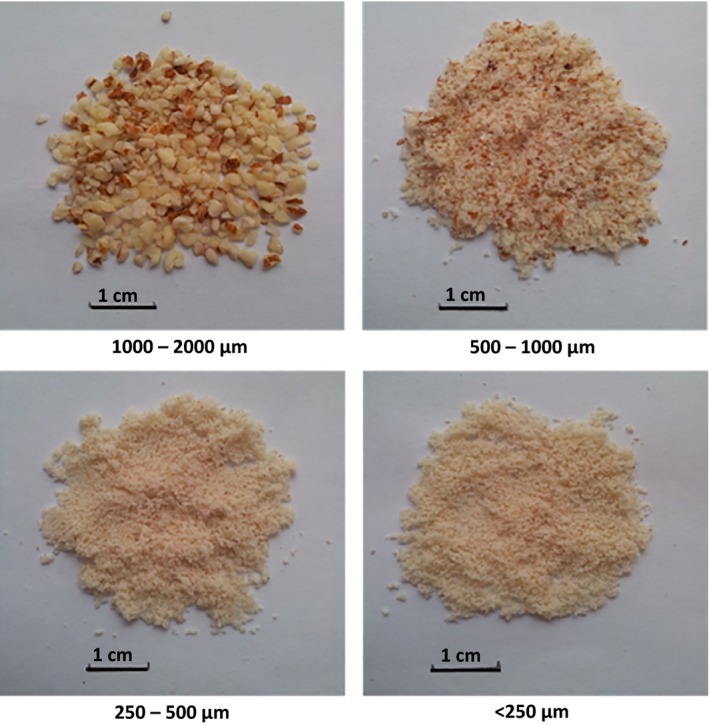
Photographs of ground almond particles with different size ranges. Scale bars = 1 cm.

Whole natural, blanched and roasted almond can be further processed to obtain almond particles of different shape and size (Fig. 3). For instance, almonds can be sliced, diced, chopped, ground or slivered (Wareing *et al*., 2000). These almond products differ in the proportion of intact and ruptured cells (Grundy *et al*., 2015b). Particles of smaller size have more fractured cells and thereby greater nutrient release (bioaccessibility) than larger particles.

Almond paste, or marzipan, is a mixture of sugar and ground almond (Gradziel, 2011). It can be eaten on its own or, more commonly, as part of confectionary and cake. Almond butter has a rich, creamy texture and can be used as an alternative to diary butter. The term ‘nut butter’ refers to a butter made from a nut, such as almond, containing at least 90% of a nut compound, which can be produced in the form of particles (chunk and/or flour), paste, oil or a combination thereof (Wilkes, 2012; Gorrepati *et al*., 2015). Almond butter is obtained from either raw or roasted almonds, with or without their skins.

### Homogenisation and oil extraction

Almond milk can be used as a plant‐based alternative to cow's milk for individuals suffering from lactose intolerance and allergy to cow milk proteins. Almond milk is a colloidal dispersion obtained by the physical disintegration, such as grinding, of almond kernels with water. Commercially available almond milk is often submitted to high pressure and heat treatment, which has consequences on its physical properties (i.e. particles/droplet sizes, rheology and protein structure) and therefore its stability (Bernat *et al*., 2015), but also on the allergenic potential of almond proteins (Dhakal *et al*., 2014). Therefore, even though the microstructure of the oil bodies within the almond milk is intact following grinding (Gallier *et al*., 2014), it appears that the monolayer of phospholipids and proteins is disrupted during the subsequent heat treatment (Bernat *et al*., 2015).

Almond oil is usually extracted by applying cold pressing to the almond kernels (Kamal‐Eldin & Moreau, 2009). Solvent or supercritical fluid extractions are other methods used to extract the oil. The oil yield is higher with these chemical extraction techniques, but the quality of the oil (i.e. purity and presence of micronutrients) is lower than the ones obtained by cold‐press. The cold‐pressed almond oil has a light and pale amber colour (Almond Board of California, 2013). During the extraction, the oil bodies completely lose their integrity. The vitamin E and phenolic compounds contained in the oil inhibit its oxidation.

### Effects of processing and storage on almond quality

Almonds that are consumed or used in the raw form (not roasted or blanched) are required to be pasteurised in the USA to remove any contaminant, in particular bacteria, mould and fungi. A water activity below 0.65 (~6% moisture content) is required to prevent growth of microorganisms when the almond is stored (Harris & Ferguson, 2013).

Lipid oxidation results from the breakdown of lipid either by enzymic activity or reaction with the atmospheric oxygen (Lin *et al*., 2012). Therefore, exposure to light and elevation in moisture content can lead to lipid oxidation. The process is minimal in almonds when the water activity is in the range of 0.25 and 0.35 (~3–4% moisture content) (Almond Board of California, 2014). Processing will have an impact on the moisture content of the almond and could promote lipid oxidation. This could be prevented by using low temperature and low humidity storage conditions (Lin *et al*., 2012).

The techniques and conditions employed for the processing of almond kernels, briefly described above, can affect its macro‐ and microstructures, which in turn can impact on the behaviour of almond tissue in the GI tract postingestion.

## Behaviour of whole and processed almonds in the GI tract and implications for macronutrient bioaccessibility, postprandial metabolism and gut microflora

### Digestion of whole, raw almonds

It has been previously shown that it is mainly the first outer layer of cells of almond particles that fracture by mechanical trituration or chewing, so that most of the parenchyma cells of almonds remain intact and therefore contain encapsulated lipid and protein (Ellis *et al*., 2004; Grundy *et al*., 2015a). However, in a study in ileostomy volunteers, the lipids present in the intact cells located underneath the fractured layers, appeared to ‘leach’ from the intact cells, but only after a prolonged incubation in the upper GI tract (Mandalari *et al*., 2008a). Indeed, ingested raw almonds collected from ileostomy volunteers after 12 h digestion showed cells with thicker (swollen) cell walls (~1.2 μm) than after 2 h digestion (~0.6 μm) and undigested cells (0.1–0.2 μm). This swelling of the cell wall may explain why intact cells lose lipid after longer retention times, suggesting that lipase, colipase and bile salt could diffuse into the intracellular compartment and then initiate lipolysis. However, lipase does not seem to diffuse through the intact cell walls even after prolonged incubation times (up to 20 h) as demonstrated by *in vitro* digestion experiments performed on laboratory‐separated almond cells (Grundy *et al*., 2016). Nevertheless, in small particles of masticated almond, there was some evidence of rupture and fissures in ‘damaged cells’ underlying the fractured surface and this may account for the lipid release that occurs after prolonged incubation in the GI tract (Mandalari *et al*., 2008a; Grundy *et al*., 2015a).

It has been suggested that the cell wall swelling is mainly attributed to the degradation and solubilisation of pectic compounds present in the cell wall and middle lamella, a process that potentially could increase porosity of the cells (Baron‐Epel *et al*., 1988; Femenia *et al*., 2001; Waldron *et al*., 2003). Nonetheless, it remains unclear to what extent lipolysis occurs inside almond cells and whether the lipids are able to leave the cells as TAG molecules or hydrolysed products. Even though lipase appears to be able to penetrate inside some cells, most likely the damaged ones, much of the lipid (TAG and/or lipolytic products) remains encapsulated inside the almond cells (Grundy *et al*., 2016). Regardless of the mechanism involved, the rate and extent of digestion of the lipids present in these unfractured cells is significantly reduced, as that they are less accessible to emulsification and digestion by the lipases (Grundy *et al*., 2015b).

What is very clear from these almond studies is that the cells of the almond cotyledon behave in a fairly predictable way as they fracture rather than separate after chewing (Ellis *et al*., 2004) or by mechanical processing such as cutting and milling (Grassby *et al*., 2014), most likely due to their strong cell‐cell adhesion (Waldron *et al*., 2003). Therefore, mechanical processing (mainly grinding) or mastication is necessary for the cells to rupture and allow intra‐cellular lipid and other nutrients (e.g. proteins) to be made available for digestion. The released lipids seem to coalesce and form droplets (size ~10–40 μm) at the surface of the ruptured cells, thus becoming available for lipolysis by the lipases (Ellis *et al*., 2004).

A digestion model that simulates the gastric environment provided contradictory information on the behaviour of almond particles in the digestive tract (Kong & Singh, 2009). Almond cells appeared to separate following the acidic hydrolysis of the middle lamella, which lessened the cell‐cell adhesion. The authors also detected the presence of breach and breakage in cell walls causing the release of nutrients into the extracellular environment and/or the penetration of enzyme and digestive components into the cells.

Collection of faeces after ingestion of almond kernels revealed the presence of significant amounts of almond tissues (cotyledon and testa) (Ellis *et al*., 2004). Some of the cells were found intact, whereas others contained bacteria that seemed to be utilising (i.e. fermenting) both intracellular nutrients (including lipid) and cell wall polysaccharides (notably pectic substances). Indeed, the erosion of cell walls, the presence of virtually empty cells (i.e. no intra‐cellular nutrients) and apparent bacterial replication provide some evidence for the potential role of almonds as a source of nutrients for the gut microflora. Mandalari and colleagues have confirmed the prebiotic role of almonds and that the lipid components of almonds are susceptible to fermentation (Mandalari *et al*., 2008b). Moreover, since the lipids provide most of the energy contained in the almond, undigested lipids excreted in the faeces could have an impact on energy metabolism. Evidence to support this hypothesis is provided by measurements of the metabolisable energy content of almonds in healthy human subjects (Novotny *et al*., 2012). These findings indicate that the energy values of raw almonds, calculated using the conventional Atwater factor, overestimate the amount of energy actually absorbed.

### Digestion of processed almonds

*In vitro* (Mandalari *et al*., 2008a) and *in vivo* (Berry *et al*., 2008) studies have revealed marked variations in lipolysis rates and postprandial blood TAG concentrations between meals containing different forms of almond (whole natural, blanched, milled flour and free oil), which are mainly attributed to differences in lipid release (bioaccessibility). In the oil form, lipids were highly available and therefore fully digested (leading to a high concentration of TAG in the blood), whereas encapsulated nutrients (whole almonds) did not lead to a postprandial response as rapid and strong as the almond oil (Berry *et al*., 2008). These results strengthen the assumption that by increasing the number of fractured cells through either processing or mastication, the bioaccessibility of nutrients, especially lipids, is enhanced. More recent studies have confirmed that almonds consumed as the whole kernel (raw or roasted) were not fully digested, and the lipids were released slowly during the digestion process (Grassby *et al*., 2014; Mandalari *et al*., 2014; Grundy *et al*., 2015a,b, 2016). This behaviour is strongly linked to the resistance of almond tissue/cell walls to chemical and physical breakdown in the mouth, stomach and small intestine. When the oil bodies are released from the almond tissue, as this is the case in almond milk, they are highly digestible and the rate and extent of lipolysis is similar to emulsified almond oil (Beisson *et al*., 2001a; Gallier & Singh, 2012; Grundy *et al*., 2016). If not in the form of oil bodies, almond oil, like any other edible plant oil, is required to be emulsified and its susceptibility to digestion relies on the size and interfacial quality (e.g. molecules adsorbed and surface tension) of the oil droplets (Gallier *et al*., 2014; Grundy *et al*., 2015b).

In an experiment performed in the pig, a useful animal model for studies of digestion and postprandial metabolism, no differences in plasma glucose or lipid levels were found between raw and roasted almonds (Bornhorst *et al*., 2013a). However, the same authors reported that gastric emptying of protein in pigs was more rapid for raw as compared with roasted almonds due to protein segregation. In more recent studies, it was shown that although the masticated bolus of roasted almonds contained a higher proportion of particles of small size compared with raw almond bolus (Grundy *et al*., 2015a), there were negligible differences in lipid release in the gastric compartment (Mandalari *et al*., 2014) and the time course of lipid digestion during the duodenal phase (Grundy *et al*., 2015b) between the two almond forms. Another study performed in pigs showed no difference in particle sizes and rheological behaviour between raw and roasted almonds during gastric digestion (Bornhorst *et al*., 2013b). It was also recently reported by Gallier and colleagues that there was no variation in ileal lipid digestibility in rats fed either crushed whole almonds, almond oil emulsion or almond oil bodies (Gallier *et al*., 2014). This surprising result may be ascribed to the fact that the gastric emptying rate of raw almonds was slower than almond cream and oil, leaving enough time for the almond tissue to be degraded.

Finally, quantitative and qualitative analysis of the carbohydrates that comprise the cell walls of digested, finely ground almonds revealed that they were not degraded during digestion; however, some of the intracellular content was fermented by the microorganisms originating from the human large intestine (Mandalari *et al*., 2008b). By comparing the growth of faecal bacteria cultures between almond kernels with normal lipid content and defatted ones, these authors also confirmed the assumption made by Ellis and colleagues that gut bacteria utilise almond lipids as a source of energy for growth and maintenance (Ellis *et al*., 2004).

## Conclusions

The beneficial health effects of almonds rely not only on their nutritional composition, as they are a good source of unsaturated fatty acids, vitamin E, polyphenols and phytosterols, but also on their structure and properties when ingested. Differences in the physical form of ingested almonds in particular, lead to variability in nutrient digestibility and consequently evoke different blood nutrient profiles and gut hormone responses. The potential cardioprotective effects of almonds and their high satiety value reported in the literature suggest that they would make a healthy snack, especially when consumed as whole kernels. Energy values of raw almonds calculated using Atwater factors have been shown to be an overestimate of their actual metabolisable energy. This finding together with the results from the studies presented in this review raise important nutritional questions about the validity of energy content values found on food labels, which are based on food composition data and Atwater correction factors.

## Conflict of interest

Karen Lapsley is an employee of the Almond Board of California.

## Note

1 Fruit that possesses simultaneously fleshy (mesocarp or hull) and stony (endocarp or shell) layers surrounding the kernel Armstrong, (2009).
